# The crystal structure of zwitterionic 2-{[(4-imin­iumyl-3-methyl-1,4-di­hydro­pyridin-1-yl)meth­yl]carbamo­yl}benzoate hemihydrate

**DOI:** 10.1107/S2056989017007836

**Published:** 2017-06-02

**Authors:** C. S. Chidan Kumar, Ai Jia Sim, Weng Zhun Ng, Tze Shyang Chia, Wan-Sin Loh, Huey Chong Kwong, Ching Kheng Quah, S. Naveen, N. K. Lokanath, Ismail Warad

**Affiliations:** a Department of Engineering Chemistry, Vidya Vikas Institute of Engineering & Technology, Visvesvaraya Technological University, Alanahally, Mysuru 570 028, India; b X-ray Crystallography Unit, School of Physics, Universiti Sains Malaysia, 11800 USM, Penang, Malaysia; c School of Chemical Sciences, Universiti Sains Malaysia, 11800 USM, Penang, Malaysia; d Institution of Excellence, University of Mysore, Manasagangotri, Mysuru 570 006, India; e Department of Studies in Physics, University of Mysore, Manasagangotri, Mysuru 570 006, India; f Department of Chemistry, Science College, An-Najah National University, PO Box 7, Nablus, West Bank, Palestinian Territories

**Keywords:** crystal structure, zwitterion, hydrogen bonding, π–π inter­actions

## Abstract

The asymmetric unit of the title compound, C_15_H_15_N_3_O_3_·0.5H_2_O, comprises two 2-{[(4-iminiumyl-3-methyl-1,4-di­hydro­pyridin-1-yl)meth­yl]carbamo­yl}benzoate zwitterions (*A* and *B*) and a water mol­ecule. The dihedral angles between the pyridine and phenyl rings in the zwitterions are 53.69 (10) and 73.56 (11)° in *A* and *B*, respectively. In the crystal, mol­ecules are linked by N—H⋯O, O—H⋯O, C—H⋯O and C—H⋯π(ring) hydrogen bonds into a three-dimensional network. The crystal structure also features π–π inter­actions involving the centroids of the pyridine and phenyl rings [centroid–centroid distances = 3.5618 (12) Å in *A* and 3.8182 (14) Å in *B*].

## Abstract 2

The mol­ecular and crystal structure of zwitterionic 2-{[(4-iminiumyl-3-methyl-1,4-di­hydro­pyridin-1-yl)meth­yl]carbamo­yl}benzoate hemihydrate is reported. The crystal structure is stabilized by a variety of hydrogen bonds and offset π–π stacking inter­actions.

## Chemical context   

Zwitterions are high-performance materials that can be used as drug protein stabilizers without affecting the activity of the drug (Keefe & Jiang, 2012 ▸). Drug protein stabilizers not only maintain the native chemical structure, but the native secondary and higher order structures necessary for biological activity and can increase the stability of the therapeutic protein and enhancs protein–substrate hydrophobic interactions without affecting the activity of the drugs. Zwitterionic polymers grafted from polysulfone (PSF) membranes show improved protein anti-­fouling properties, together with good blood compatibility and cytocompatibility in comparison with the pristine PSF membrane (Yue *et al.*, 2013 ▸). Furthermore, zwitterionic nanocarrier drugs showed excellent biocompatibility and non-fouling properties, and were found to extend blood circulation times *in vivo*. The study and synthesis of new zwitterions is therefore important in the search for new biomedical applications (Jin *et al.*, 2014 ▸).
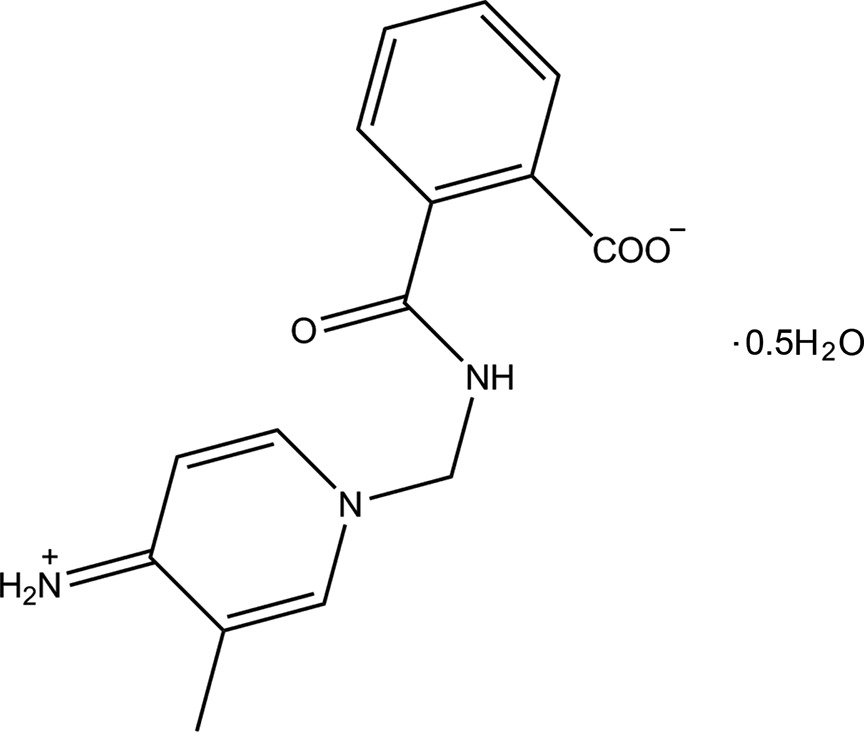


## Structural commentary   

**Figure 1:**
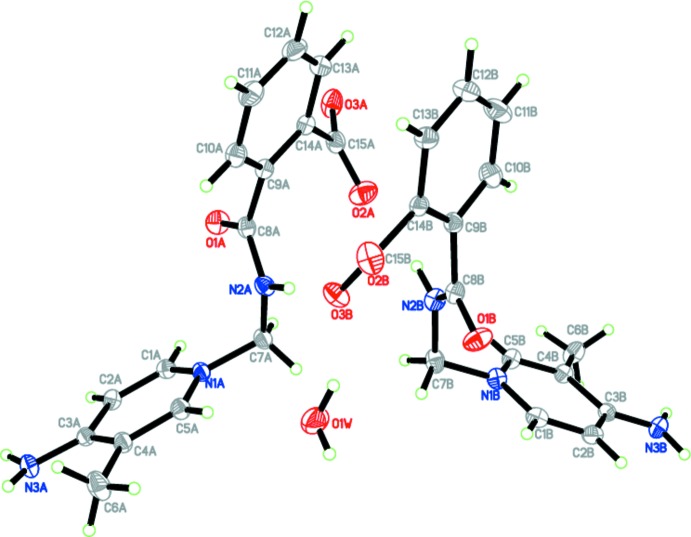
The mol­ecular structure of the title compound, with the atom labelling and 50% probability displacement ellipsoids.

The asymmetric unit of the title compound comprises two crystallographically independent 2-{[(4-iminiumyl-3-methyl-1,4-di­hydro­pyridin-1-yl)meth­yl]carbamo­yl}benzoate zwitterions (mol­ecules *A* and *B*) and a cocrystallized water mol­ecule, as shown in Fig. 1 ▸. The zwitterions are formed through protonation of the imine substituent on the pyridine ring and deprotonation of the carboxyl­ate substituent on the benzene ring. The bond lengths and angles (Table 1 ▸) in the title compound (Fig. 1 ▸) are generally within normal ranges. However, the N3—C3 [1.335 (3) Å in both molecules] are shorter than expected for an NH_2_—C_ar_ single bond [1.38 (3) Å], but are similar to those found in related compounds with an N^+^=C double bond (Sharmila *et al.*, 2014 ▸; Sun *et al.*, 2015 ▸). The C—O bonds in the carboxyl­ate units [C15*A*—O3*A* = 1.247 (3) Å and C15*A*—O2*A* = 1.257 (3) Å] in mol­ecule *A*, with comparable values in mol­ecule *B*, are similar to values found in other deprotonated carboxyl­ate groups (Hemamalini & Fun, 2010 ▸).

## Supra­molecular features   

**Figure 2:**
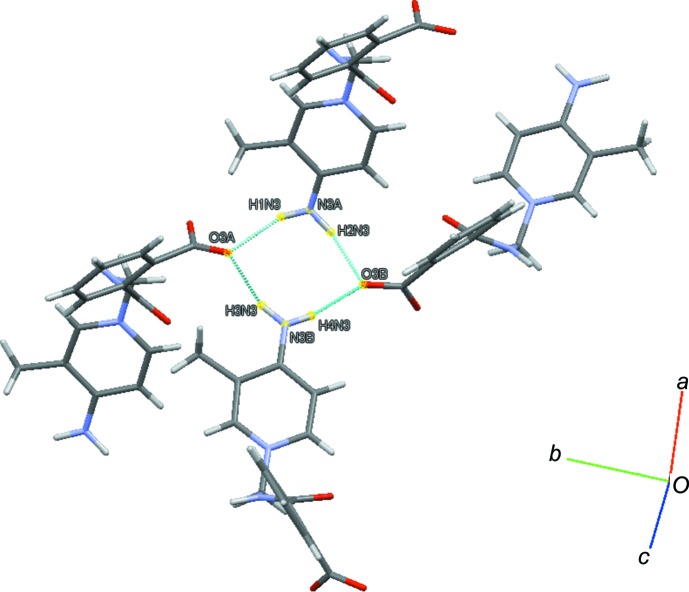
A partial packing diagram, with an 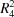
(8) ring motif generated by N—H⋯O hydrogen bonds (dotted lines).

**Figure 3:**
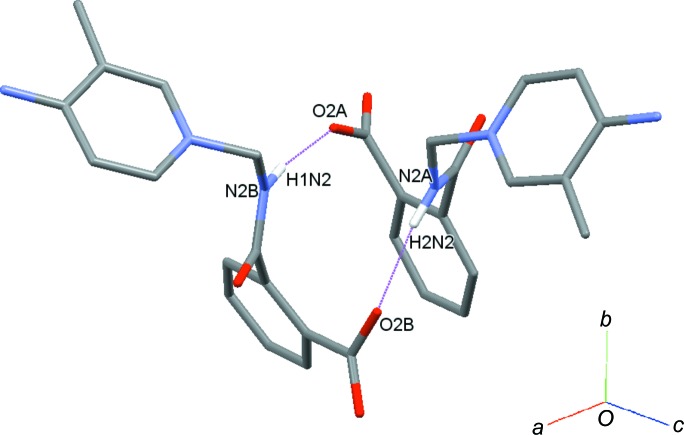
A dimer with an 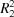
(14) ring motif generated by N—H⋯O hydrogen bonds (dotted lines).

**Figure 4:**
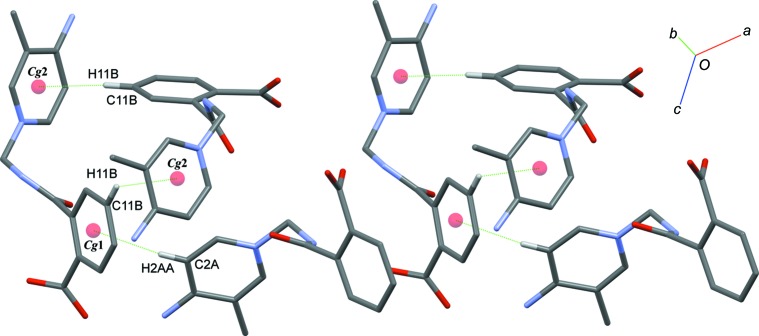
A partial packing diagram of the title compound, with C—H⋯π inter­actions (dotted lines).

**Figure 5:**
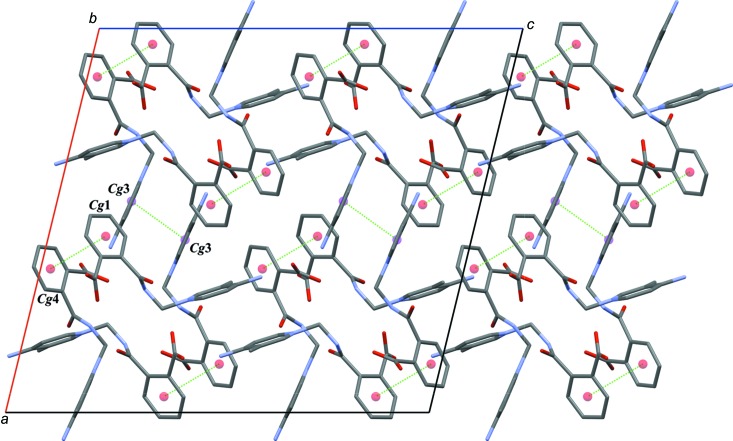
The mol­ecular packing in the title compound with two kinds of π–π inter­actions (dotted lines).

**Figure 6:**
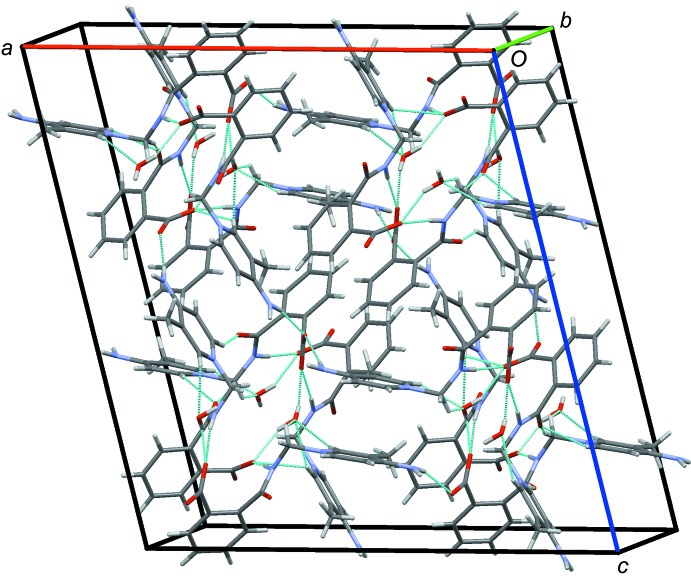
The overall packing of the title compound, viewed along the *b*-axis direction, showing parallel sheets in the *ac* plane linked into a three-dimensional network along *b*.

In the crystal, mol­ecules are linked by N—H⋯O, C—H⋯O and O—H⋯O hydrogen bonds (Table 2 ▸) into a three-dimensional network. Inter­molecular N3*A*—H1*N*3⋯O3*A*, N3*A*—H2*N*3⋯O3*B*, N3*B*—H3*N*3⋯O3*A* and N3*B*—H4*N*3⋯O3*B* hydrogen bonds generate 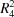
(8) ring motifs (Fig. 2 ▸), while N2*A*—H2*N*2⋯O2*B* and N2*B*—H1*N*2⋯O2*A* hydrogen bonds form dimers with 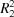
(14) ring motifs (Fig. 3 ▸). Mol­ecule *A* is connected to mol­ecule *B* through a C2*A*—H2*AA*⋯*Cg*1 inter­action, while mol­ecules of *B* are linked by C11*B*—H11*B*⋯*Cg*2 inter­actions (*Cg*1 and *Cg*2 are the centroids of the C9*B*–C14*B* and N1*B*/C1*B*–C5*B* rings) (Fig. 4 ▸). The crystal structure also features π–π inter­actions [*Cg*3⋯*Cg*3(−*x*, *y*, −*z* + 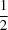) = 3.5618 (12) Å; *Cg*1⋯*Cg*4 = 3.8182 (14) Å, where *Cg*3 and *Cg*4 are the centroids of the N1*A*/C1*A*–C5*A* and C9*A*–C14*A* rings] (Fig. 5 ▸). An overall packing diagram, showing the three-dimensional array of parallel sheet of mol­ecules in the *ac* plane is shown in Fig. 6 ▸.

## Database survey   

Eight structures containing carbamoylbenzoates were found in a search of the Cambridge Structural Database (Version 3.57; Groom *et al.*, 2016 ▸): *N*-[2-(4,5,6,7-tetra­hydro­benz­imid­azol-2-­yl)eth­yl]phthalamic acid tetra­hydrate (Elz *et al.*, 1983 ▸), 2-(phenyl­carbamo­yl)benzoic acid (Smith *et al.*, 1983 ▸), *N*-(4-chloro­phen­yl)phthalamic acid (Mornon, 1970 ▸), 2-(pyridin-4-ylcarbamo­yl)benzoate 4-amino­pyridinium monohydrate (Zhu *et al.*, 2010 ▸), phthalimide–phthalamate monohydrate (Barrett *et al.*, 1998 ▸), bi­cyclo­[2.2.1]heptan-2-aminium (*R*)-2-[(1-phenyl­eth­yl)carbamo­yl]benzoate (Caille *et al.*, 2009 ▸), bis­(tri­methyl­ammonium) 7-[2-(carboxyl­ato)benzamido­eth­yl]-7,8-di­carba-*nido*-undeca­borate(10) (Batsanov *et al.*, 2001 ▸) and (*R*)-1-phenyl­ethanaminium 2-{[(2*R*,3*R*)-2,3-dimeth­oxy-2,3-di­methyl-2,3-di­hydro-1,4-benzodioxin-6-yl]carbamo­yl}benzoate (Ramarao *et al.*, 2012 ▸).

A search for imino­pyridine derivatives using 4-(λ^4^-aza­nyl­idene)-4*H*-1λ^2^-pyridine as the skeleton gave 15 hits, although none of these were zwitterionic derivatives comparable to the title compound. Of these, only three had aromatic rings in the cation in addition to the imino­poyridine unit (Sharmila *et al.*, 2014 ▸; Pei *et al.*, 2013 ▸)

## Synthesis and crystallization   

The title compound was obtained unexpectedly from the reaction of 0.01 mol of *N*-(bromo­meth­yl)phthalimide and 0.01 mol of 4-amino-3-methyl­pyridine in 10 ml of di­methyl­formamide with a catalytic amount of potassium carbonate. The mixture was stirred in a 50 ml round-bottomed flask at room temperature for about 3 h. The progress of the reaction was monitored by thin-layer chromatography and the mixture was poured into cold water once the reaction was complete. The resulting precipitate was filtered off, washed successively with distilled water, and recrystallized from acetone solution by slow evaporation to obtain colourless block-shaped single crystals.

## Refinement   

Crystal data, data collection and structure refinement details are summarized in Table 3 ▸. The N- and O- bound H atoms were located from difference Fourier maps and the former were refined freely [N—H = 0.88 (2)–0.95 (3) Å], whereas for the latter, the distances from atom O1*W* were fixed at 0.86 Å, the H⋯H distance was fixed at 1.34 Å and the H atoms were refined with a riding model [*U*_iso_(H) = 1.5*U*_eq_(O), and O—H = 0.864 and 0.865 Å]. The C-bound H atoms were positioned geometrically using a riding model, with *U*_iso_(H) = 1.2 or 1.5*U*_eq_(C) (C—H = 0.93, 0.96 and 0.97 Å). A rotating-group model was applied to the methyl groups.

## Supplementary Material

Crystal structure: contains datablock(s) global, I. DOI: 10.1107/S2056989017007836/sj5531sup1.cif

Structure factors: contains datablock(s) I. DOI: 10.1107/S2056989017007836/sj5531Isup2.hkl

CCDC reference: 1552520

Additional supporting information:  crystallographic information; 3D view; checkCIF report

## Figures and Tables

**Table 1:** Selected geometric parameters (Å, °)

O1*A*—C8*A*	1.220 (3)	O1*B*—C8*B*	1.222 (3)
O2*A*—C15*A*	1.257 (3)	O2*B*—C15*B*	1.244 (3)
O3*A*—C15*A*	1.247 (3)	O3*B*—C15*B*	1.249 (3)
N1*A*—C1*A*	1.346 (3)	N1*B*—C7*B*	1.484 (3)
N1*A*—C7*A*	1.480 (3)	N1*B*—C1*B*	1.341 (3)
N1*A*—C5*A*	1.355 (3)	N1*B*—C5*B*	1.351 (3)
N2*A*—C8*A*	1.353 (3)	N2*B*—C7*B*	1.435 (3)
N2*A*—C7*A*	1.430 (3)	N2*B*—C8*B*	1.349 (3)
N3*A*—C3*A*	1.335 (3)	N3*B*—C3*B*	1.335 (3)
			
C1*A*—N1*A*—C5*A*	119.30 (18)	C1*B*—N1*B*—C5*B*	119.32 (18)
C5*A*—N1*A*—C7*A*	119.47 (17)	C1*B*—N1*B*—C7*B*	120.78 (18)
C1*A*—N1*A*—C7*A*	121.19 (17)	C5*B*—N1*B*—C7*B*	119.84 (17)
C7*A*—N2*A*—C8*A*	119.71 (18)	C7*B*—N2*B*—C8*B*	120.11 (19)
N1*A*—C1*A*—C2*A*	121.03 (19)	N1*B*—C1*B*—C2*B*	121.2 (2)
N3*A*—C3*A*—C2*A*	121.5 (2)	N3*B*—C3*B*—C2*B*	122.2 (2)
N3*A*—C3*A*—C4*A*	121.5 (2)	N3*B*—C3*B*—C4*B*	120.95 (19)
N1*A*—C5*A*—C4*A*	123.08 (19)	N1*B*—C5*B*—C4*B*	123.12 (19)
N1*A*—C7*A*—N2*A*	113.36 (16)	N1*B*—C7*B*—N2*B*	113.19 (19)
O1*A*—C8*A*—N2*A*	121.8 (2)	O1*B*—C8*B*—C9*B*	121.33 (19)
N2*A*—C8*A*—C9*A*	116.71 (18)	N2*B*—C8*B*—C9*B*	116.39 (18)
O1*A*—C8*A*—C9*A*	121.54 (18)	O1*B*—C8*B*—N2*B*	122.1 (2)
O3*A*—C15*A*—C14*A*	117.7 (2)	O2*B*—C15*B*—C14*B*	117.17 (17)
O2*A*—C15*A*—O3*A*	126.7 (2)	O2*B*—C15*B*—O3*B*	125.5 (2)
O2*A*—C15*A*—C14*A*	115.5 (2)	O3*B*—C15*B*—C14*B*	117.32 (19)

**Table 2:** Hydrogen-bond geometry (Å, °) *Cg*1 and *Cg*2 are the centroids of the C9*B*–C14*B* and N1*B*/C1*B*–C5*B* rings, respectively.

*D*—H⋯*A*	*D*—H	H⋯*A*	*D*⋯*A*	*D*—H⋯*A*
N2*A*—H2*N*2⋯O2*B*	0.88 (2)	2.03 (2)	2.897 (2)	173 (2)
N3*A*—H2*N*3⋯O3*B* ^i^	0.93 (3)	1.95 (3)	2.864 (3)	168 (2)
N3*A*—H1*N*3⋯O3*A* ^ii^	0.90 (2)	2.01 (3)	2.864 (3)	157 (2)
N2*B*—H1*N*2⋯O2*A*	0.90 (3)	2.12 (3)	3.006 (3)	171 (3)
N3*B*—H4*N*3⋯O3*B* ^iii^	0.89 (2)	2.01 (3)	2.887 (3)	167 (2)
N3*B*—H3*N*3⋯O3*A* ^iv^	0.95 (3)	2.03 (3)	2.935 (3)	161 (3)
O1*W*—H1*W*1⋯O2*B*	0.87	1.87	2.681 (3)	155
O1*W*—H2*W*1⋯O2*A* ^v^	0.86	1.81	2.581 (3)	147
C1*A*—H1*AA*⋯O1*B* ^vi^	0.93	2.53	3.355 (3)	147
C5*A*—H5*AA*⋯O1*W*	0.93	2.27	3.083 (3)	146
C7*A*—H7*AA*⋯O1*W*	0.97	2.47	3.139 (3)	126
C7*A*—H7*AB*⋯O1*B* ^vi^	0.97	2.40	3.336 (3)	162
C1*B*—H1*BA*⋯O1*A* ^v^	0.93	2.20	3.053 (3)	152
C2*A*—H2*AA*⋯*Cg*1^i^	0.93	2.95	3.831 (2)	158
C11*B*—H11*B*⋯*Cg*2^vii^	0.93	2.94	3.721 (3)	142

Symmetry codes: (i) 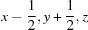; (ii) 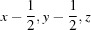; (iii) 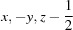; (iv) 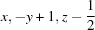; (v) 
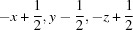
; (vi) 
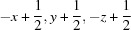
; (vii) 
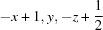
.

**Table 3:** Experimental details

Crystal data	
Chemical formula	C_15_H_15_N_3_O_3_·0.5H_2_O
*M*_r_	294.31
Crystal system, space group	Monoclinic, *C*2/*c*
Temperature (K)	294
*a*, *b*, *c* (Å)	21.3157 (18), 11.9883 (8), 22.8642 (15)
β (°)	103.729 (2)
*V* (Å^3^)	5675.8 (7)
*Z*	16
Radiation type	Mo *K*α
μ (mm^−1^)	0.10
Crystal size (mm)	0.28 × 0.26 × 0.13

Data collection	
Diffractometer	Bruker APEXII DUO CCD area-detector
Absorption correction	Multi-scan (*SADABS*, 2012 ▸)
*T*_min_, *T*_max_	0.962, 0.996
No. of measured, independent and observed [*I* > 2σ(*I*)] reflections	65196, 5430, 3849
*R*_int_	0.052
(sin θ/λ)_max_ (Å^−1^)	0.613

Refinement	
*R*[*F* ^2^ > 2σ(*F* ^2^)], *wR*(*F* ^2^), *S*	0.047, 0.136, 1.04
No. of reflections	5430
No. of parameters	414
H-atom treatment	H atoms treated by a mixture of independent and constrained refinement
Δρ_max_, Δρ_min_ (e Å^−3^)	0.48, −0.29

Computer programs: *APEX2* and *SAINT* (Bruker, 2012 ▸), *SHELXS97* (Sheldrick, 2008 ▸), *SHELXL2013* (Sheldrick, 2015 ▸), *Mercury* (Macrae *et al.*, 2006 ▸) and *PLATON* (Spek, 2009 ▸).

## supplementary crystallographic information

### Crystal data

**Table:**

C_15_H_15_N_3_O_3_·0.5H_2_O	*F*(000) = 2480
*M**_r_* = 294.31	*D*_x_ = 1.378 Mg m^−^^3^
Monoclinic, *C*2/*c*	Mo *K*α radiation, λ = 0.71073 Å
Hall symbol: -C 2yc	Cell parameters from 8600 reflections
*a* = 21.3157 (18) Å	θ = 2.4–21.2°
*b* = 11.9883 (8) Å	µ = 0.10 mm^−^^1^
*c* = 22.8642 (15) Å	*T* = 294 K
β = 103.729 (2)°	Block, colourless
*V* = 5675.8 (7) Å^3^	0.28 × 0.26 × 0.13 mm
*Z* = 16	

### Data collection

**Table:**

Bruker APEXII DUO CCD area-detector diffractometer	5430 independent reflections
Radiation source: Rotating Anode	3849 reflections with *I* > 2σ(*I*)
Graphite monochromator	*R*_int_ = 0.052
Detector resolution: 18.4 pixels mm^-1^	θ_max_ = 25.8°, θ_min_ = 1.8°
φ and ω scans	*h* = −25→25
Absorption correction: multi-scan SADABS 2014/5	*k* = −14→14
*T*_min_ = 0.962, *T*_max_ = 0.996	*l* = −27→27
65196 measured reflections	

### Refinement

**Table:**

Refinement on *F*^2^	0 restraints
Least-squares matrix: full	Hydrogen site location: mixed
*R*[*F*^2^ > 2σ(*F*^2^)] = 0.047	H atoms treated by a mixture of independent and constrained refinement
*wR*(*F*^2^) = 0.136	W = 1/[Σ^2^(*F*O^2^) + (0.0647*P*)^2^ + 3.8981*P*] where *P* = (*F*O^2^ + 2*F*C^2^)/3
*S* = 1.04	(Δ/σ)_max_ < 0.001
5430 reflections	Δρ_max_ = 0.48 e Å^−^^3^
414 parameters	Δρ_min_ = −0.29 e Å^−^^3^

### Special details

**Table:**

Geometry. Bond distances, angles etc. have been calculated using the rounded fractional coordinates. All su's are estimated from the variances of the (full) variance-covariance matrix. The cell esds are taken into account in the estimation of distances, angles and torsion angles
Refinement. Refinement on F^2^ for ALL reflections except those flagged by the user for potential systematic errors. Weighted R-factors wR and all goodnesses of fit S are based on F^2^, conventional R-factors R are based on F, with F set to zero for negative F^2^. The observed criterion of F^2^ > 2sigma(F^2^) is used only for calculating -R-factor-obs etc. and is not relevant to the choice of reflections for refinement. R-factors based on F^2^ are statistically about twice as large as those based on F, and R-factors based on ALL data will be even larger.

### Fractional atomic coordinates and isotropic or equivalent isotropic displacement parameters (Å^2^)

**Table:**

	*x*	*y*	*z*	*U*_iso_*/*U*_eq_	
O1A	0.22583 (8)	0.40107 (12)	0.40032 (7)	0.0547 (5)	
O2A	0.34333 (10)	0.40853 (15)	0.33565 (7)	0.0715 (7)	
O3A	0.38535 (9)	0.53318 (14)	0.40709 (8)	0.0686 (7)	
N1A	0.11034 (8)	0.25855 (14)	0.31440 (7)	0.0374 (5)	
N2A	0.22652 (8)	0.23844 (15)	0.35155 (8)	0.0410 (6)	
N3A	−0.06918 (9)	0.2157 (2)	0.34758 (9)	0.0475 (7)	
C1A	0.07352 (10)	0.34764 (17)	0.31936 (9)	0.0411 (7)	
C2A	0.01405 (10)	0.33503 (17)	0.32906 (9)	0.0409 (7)	
C3A	−0.01122 (10)	0.22851 (17)	0.33576 (8)	0.0379 (6)	
C4A	0.02754 (10)	0.13531 (17)	0.32905 (9)	0.0414 (7)	
C5A	0.08670 (10)	0.15470 (17)	0.31853 (9)	0.0422 (7)	
C6A	0.00423 (13)	0.01857 (19)	0.33375 (14)	0.0650 (10)	
C7A	0.17511 (10)	0.27198 (19)	0.30229 (9)	0.0433 (7)	
C8A	0.25260 (10)	0.31309 (17)	0.39486 (9)	0.0395 (7)	
C9A	0.31568 (10)	0.28213 (16)	0.43608 (9)	0.0384 (6)	
C10A	0.32083 (12)	0.19564 (18)	0.47694 (10)	0.0488 (8)	
C11A	0.37896 (13)	0.1736 (2)	0.51714 (10)	0.0577 (9)	
C12A	0.43176 (13)	0.2375 (2)	0.51666 (10)	0.0598 (9)	
C13A	0.42725 (11)	0.3252 (2)	0.47687 (10)	0.0521 (8)	
C14A	0.36955 (10)	0.34807 (18)	0.43589 (9)	0.0414 (7)	
C15A	0.36504 (11)	0.43856 (19)	0.38932 (11)	0.0494 (8)	
O1B	0.33946 (9)	0.04329 (13)	0.23061 (7)	0.0606 (6)	
O2B	0.29329 (7)	0.02903 (12)	0.34775 (7)	0.0534 (6)	
O3B	0.35853 (8)	−0.10987 (13)	0.38676 (8)	0.0601 (6)	
N1B	0.29345 (8)	0.24185 (14)	0.13343 (8)	0.0420 (6)	
N2B	0.32218 (9)	0.22607 (16)	0.24284 (8)	0.0446 (6)	
N3B	0.35284 (10)	0.27772 (19)	−0.02411 (9)	0.0498 (7)	
C1B	0.29850 (10)	0.15205 (18)	0.09987 (10)	0.0450 (7)	
C2B	0.31782 (10)	0.16157 (17)	0.04763 (10)	0.0443 (7)	
C3B	0.33333 (9)	0.26616 (17)	0.02691 (9)	0.0391 (6)	
C4B	0.32668 (10)	0.36049 (16)	0.06271 (9)	0.0401 (7)	
C5B	0.30681 (10)	0.34382 (17)	0.11433 (9)	0.0424 (7)	
C6B	0.33867 (14)	0.47561 (19)	0.04276 (11)	0.0590 (9)	
C7B	0.27054 (11)	0.2309 (2)	0.18961 (10)	0.0491 (8)	
C8B	0.35212 (10)	0.12839 (17)	0.26047 (9)	0.0410 (7)	
C9B	0.40518 (10)	0.13125 (17)	0.31648 (9)	0.0399 (6)	
C10B	0.45840 (12)	0.1987 (2)	0.31831 (12)	0.0596 (9)	
C11B	0.51180 (13)	0.1928 (3)	0.36621 (14)	0.0736 (11)	
C12B	0.51240 (12)	0.1204 (2)	0.41257 (13)	0.0659 (10)	
C13B	0.45962 (11)	0.0541 (2)	0.41153 (10)	0.0515 (8)	
C14B	0.40539 (9)	0.05834 (16)	0.36407 (9)	0.0371 (6)	
C15B	0.34765 (10)	−0.01351 (16)	0.36602 (9)	0.0386 (7)	
O1W	0.18224 (9)	0.0223 (2)	0.26300 (9)	0.0921 (9)	
H2N2	0.2484 (10)	0.1778 (19)	0.3480 (9)	0.036 (6)*	
H2N3	−0.0890 (14)	0.279 (2)	0.3580 (12)	0.074 (9)*	
H1N3	−0.0822 (13)	0.148 (2)	0.3573 (12)	0.071 (8)*	
H1AA	0.08940	0.41900	0.31600	0.0490*	
H2AA	−0.01080	0.39790	0.33140	0.0490*	
H5AA	0.11220	0.09380	0.31390	0.0510*	
H6AA	−0.03720	0.00910	0.30630	0.0980*	
H6AB	0.00040	0.00490	0.37410	0.0980*	
H6AC	0.03450	−0.03310	0.32380	0.0980*	
H7AA	0.17720	0.22820	0.26710	0.0520*	
H10A	0.28490	0.15200	0.47740	0.0590*	
H7AB	0.18120	0.34960	0.29310	0.0520*	
H11A	0.38210	0.11520	0.54450	0.0690*	
H12A	0.47100	0.22190	0.54330	0.0720*	
H13A	0.46330	0.36940	0.47750	0.0630*	
H10B	0.45820	0.24840	0.28700	0.0720*	
H11B	0.54740	0.23820	0.36690	0.0880*	
H1N2	0.3306 (14)	0.285 (3)	0.2678 (14)	0.086 (10)*	
H12B	0.54850	0.11610	0.44470	0.0790*	
H4N3	0.3539 (12)	0.218 (2)	−0.0473 (12)	0.061 (7)*	
H13B	0.46030	0.00530	0.44330	0.0620*	
H3N3	0.3629 (13)	0.348 (3)	−0.0382 (12)	0.077 (9)*	
H1BA	0.28860	0.08200	0.11260	0.0540*	
H2BA	0.32090	0.09800	0.02520	0.0530*	
H5BA	0.30220	0.40550	0.13760	0.0510*	
H6BA	0.31080	0.48980	0.00390	0.0890*	
H6BB	0.38290	0.48200	0.04030	0.0890*	
H6BC	0.33010	0.52890	0.07120	0.0890*	
H7BA	0.24300	0.29390	0.19280	0.0590*	
H7BB	0.24470	0.16370	0.18730	0.0590*	
H1W1	0.22220	0.01410	0.28200	0.1380*	
H2W1	0.17600	−0.03640	0.24040	0.1380*	

### Atomic displacement parameters (Å^2^)

**Table:**

	*U*^11^	*U*^22^	*U*^33^	*U*^12^	*U*^13^	*U*^23^
O1A	0.0601 (10)	0.0382 (8)	0.0649 (10)	0.0109 (7)	0.0128 (8)	−0.0017 (7)
O2A	0.0976 (14)	0.0641 (11)	0.0488 (10)	−0.0138 (10)	0.0096 (9)	0.0080 (8)
O3A	0.0942 (14)	0.0495 (10)	0.0758 (12)	−0.0161 (9)	0.0477 (10)	−0.0041 (9)
N1A	0.0341 (9)	0.0401 (9)	0.0390 (9)	−0.0023 (7)	0.0105 (7)	0.0026 (7)
N2A	0.0343 (9)	0.0373 (10)	0.0516 (11)	0.0018 (8)	0.0104 (8)	−0.0016 (8)
N3A	0.0398 (11)	0.0490 (12)	0.0581 (12)	0.0002 (9)	0.0204 (9)	0.0038 (10)
C1A	0.0438 (12)	0.0375 (11)	0.0418 (11)	−0.0019 (9)	0.0099 (9)	0.0025 (9)
C2A	0.0434 (12)	0.0381 (11)	0.0424 (11)	0.0060 (9)	0.0127 (9)	0.0021 (9)
C3A	0.0367 (11)	0.0444 (11)	0.0323 (10)	−0.0001 (9)	0.0074 (8)	0.0020 (8)
C4A	0.0411 (12)	0.0400 (11)	0.0452 (12)	−0.0026 (9)	0.0143 (9)	−0.0010 (9)
C5A	0.0411 (12)	0.0389 (11)	0.0480 (12)	0.0040 (9)	0.0133 (9)	−0.0003 (9)
C6A	0.0618 (16)	0.0428 (13)	0.100 (2)	−0.0044 (12)	0.0381 (15)	0.0023 (13)
C7A	0.0366 (11)	0.0497 (12)	0.0452 (12)	−0.0024 (9)	0.0129 (9)	0.0045 (9)
C8A	0.0425 (12)	0.0339 (11)	0.0455 (11)	−0.0015 (9)	0.0171 (9)	0.0052 (9)
C9A	0.0413 (11)	0.0391 (11)	0.0365 (10)	0.0014 (9)	0.0127 (9)	−0.0008 (8)
C10A	0.0564 (14)	0.0443 (12)	0.0490 (13)	0.0002 (10)	0.0192 (11)	0.0044 (10)
C11A	0.0724 (17)	0.0582 (15)	0.0419 (12)	0.0077 (13)	0.0126 (12)	0.0107 (11)
C12A	0.0611 (16)	0.0770 (17)	0.0366 (12)	0.0135 (14)	0.0022 (11)	−0.0013 (12)
C13A	0.0444 (13)	0.0623 (15)	0.0491 (13)	−0.0056 (11)	0.0099 (10)	−0.0072 (11)
C14A	0.0432 (12)	0.0459 (12)	0.0370 (11)	0.0001 (9)	0.0134 (9)	−0.0022 (9)
C15A	0.0519 (14)	0.0446 (13)	0.0573 (14)	−0.0082 (10)	0.0242 (11)	−0.0001 (11)
O1B	0.0794 (12)	0.0455 (9)	0.0485 (9)	0.0102 (8)	−0.0015 (8)	−0.0085 (7)
O2B	0.0394 (9)	0.0426 (9)	0.0765 (11)	0.0006 (7)	0.0102 (8)	0.0035 (8)
O3B	0.0658 (11)	0.0423 (9)	0.0775 (11)	0.0070 (8)	0.0277 (9)	0.0203 (8)
N1B	0.0431 (10)	0.0390 (10)	0.0444 (10)	0.0052 (8)	0.0116 (8)	0.0069 (8)
N2B	0.0538 (11)	0.0369 (10)	0.0439 (10)	0.0061 (8)	0.0133 (9)	0.0066 (8)
N3B	0.0584 (12)	0.0475 (12)	0.0461 (11)	0.0049 (10)	0.0176 (9)	−0.0073 (10)
C1B	0.0418 (12)	0.0378 (12)	0.0522 (13)	0.0000 (9)	0.0046 (10)	0.0054 (10)
C2B	0.0418 (12)	0.0376 (11)	0.0492 (12)	0.0057 (9)	0.0025 (10)	−0.0055 (9)
C3B	0.0315 (10)	0.0432 (11)	0.0405 (11)	0.0067 (9)	0.0044 (9)	−0.0004 (9)
C4B	0.0432 (12)	0.0358 (11)	0.0412 (11)	0.0028 (9)	0.0100 (9)	0.0011 (9)
C5B	0.0483 (12)	0.0362 (11)	0.0433 (11)	0.0045 (9)	0.0120 (9)	0.0030 (9)
C6B	0.0861 (18)	0.0434 (13)	0.0516 (14)	−0.0042 (12)	0.0246 (13)	−0.0004 (10)
C7B	0.0484 (13)	0.0529 (13)	0.0495 (13)	0.0062 (10)	0.0189 (11)	0.0153 (10)
C8B	0.0494 (12)	0.0362 (11)	0.0401 (11)	0.0022 (9)	0.0161 (9)	0.0025 (9)
C9B	0.0391 (11)	0.0382 (11)	0.0443 (11)	0.0002 (9)	0.0140 (9)	−0.0035 (9)
C10B	0.0575 (15)	0.0606 (15)	0.0652 (16)	−0.0133 (12)	0.0235 (13)	0.0054 (12)
C11B	0.0470 (15)	0.080 (2)	0.091 (2)	−0.0225 (14)	0.0111 (14)	−0.0056 (16)
C12B	0.0423 (14)	0.0819 (19)	0.0674 (17)	−0.0056 (13)	0.0008 (12)	−0.0084 (15)
C13B	0.0473 (13)	0.0583 (14)	0.0463 (12)	0.0031 (11)	0.0062 (10)	−0.0013 (11)
C14B	0.0378 (11)	0.0362 (10)	0.0379 (11)	0.0035 (9)	0.0105 (9)	−0.0040 (8)
C15B	0.0435 (12)	0.0343 (11)	0.0396 (11)	0.0032 (9)	0.0131 (9)	0.0008 (9)
O1W	0.0648 (12)	0.1316 (19)	0.0752 (13)	0.0212 (12)	0.0074 (10)	−0.0386 (12)

### Geometric parameters (Å, º)

**Table:**

O1A—C8A	1.220 (3)	C14A—C15A	1.507 (3)
O2A—C15A	1.257 (3)	C1A—H1AA	0.9300
O3A—C15A	1.247 (3)	C2A—H2AA	0.9300
O1W—H1W1	0.8700	C5A—H5AA	0.9300
O1W—H2W1	0.8600	C6A—H6AB	0.9600
N1A—C1A	1.346 (3)	C6A—H6AA	0.9600
N1A—C7A	1.480 (3)	C6A—H6AC	0.9600
N1A—C5A	1.355 (3)	C7A—H7AB	0.9700
N2A—C8A	1.353 (3)	C7A—H7AA	0.9700
N2A—C7A	1.430 (3)	C10A—H10A	0.9300
N3A—C3A	1.335 (3)	C11A—H11A	0.9300
N2A—H2N2	0.88 (2)	C12A—H12A	0.9300
N3A—H1N3	0.90 (2)	C13A—H13A	0.9300
N3A—H2N3	0.93 (3)	C1B—C2B	1.358 (3)
O1B—C8B	1.222 (3)	C2B—C3B	1.407 (3)
O2B—C15B	1.244 (3)	C3B—C4B	1.423 (3)
O3B—C15B	1.249 (3)	C4B—C6B	1.494 (3)
N1B—C7B	1.484 (3)	C4B—C5B	1.360 (3)
N1B—C1B	1.341 (3)	C8B—C9B	1.494 (3)
N1B—C5B	1.351 (3)	C9B—C10B	1.386 (3)
N2B—C7B	1.435 (3)	C9B—C14B	1.395 (3)
N2B—C8B	1.349 (3)	C10B—C11B	1.381 (4)
N3B—C3B	1.335 (3)	C11B—C12B	1.368 (4)
N2B—H1N2	0.90 (3)	C12B—C13B	1.373 (4)
N3B—H3N3	0.95 (3)	C13B—C14B	1.386 (3)
N3B—H4N3	0.89 (2)	C14B—C15B	1.512 (3)
C1A—C2A	1.346 (3)	C1B—H1BA	0.9300
C2A—C3A	1.408 (3)	C2B—H2BA	0.9300
C3A—C4A	1.419 (3)	C5B—H5BA	0.9300
C4A—C6A	1.498 (3)	C6B—H6BB	0.9600
C4A—C5A	1.359 (3)	C6B—H6BA	0.9600
C8A—C9A	1.494 (3)	C6B—H6BC	0.9600
C9A—C10A	1.383 (3)	C7B—H7BB	0.9700
C9A—C14A	1.395 (3)	C7B—H7BA	0.9700
C10A—C11A	1.382 (4)	C10B—H10B	0.9300
C11A—C12A	1.364 (4)	C11B—H11B	0.9300
C12A—C13A	1.379 (3)	C12B—H12B	0.9300
C13A—C14A	1.385 (3)	C13B—H13B	0.9300
			
H1W1—O1W—H2W1	102.00	C12A—C11A—H11A	120.00
C1A—N1A—C5A	119.30 (18)	C13A—C12A—H12A	120.00
C5A—N1A—C7A	119.47 (17)	C11A—C12A—H12A	120.00
C1A—N1A—C7A	121.19 (17)	C14A—C13A—H13A	120.00
C7A—N2A—C8A	119.71 (18)	C12A—C13A—H13A	120.00
C7A—N2A—H2N2	119.0 (13)	C1B—N1B—C5B	119.32 (18)
C8A—N2A—H2N2	118.7 (14)	C1B—N1B—C7B	120.78 (18)
C3A—N3A—H2N3	117.3 (18)	C5B—N1B—C7B	119.84 (17)
H2N3—N3A—H1N3	119 (2)	C7B—N2B—C8B	120.11 (19)
C3A—N3A—H1N3	120.5 (18)	N1B—C1B—C2B	121.2 (2)
C8B—N2B—H1N2	119 (2)	C1B—C2B—C3B	121.04 (19)
C7B—N2B—H1N2	120 (2)	N3B—C3B—C2B	122.2 (2)
C3B—N3B—H3N3	122.2 (18)	N3B—C3B—C4B	120.95 (19)
C3B—N3B—H4N3	119.5 (17)	C2B—C3B—C4B	116.82 (18)
H4N3—N3B—H3N3	118 (2)	C3B—C4B—C5B	118.46 (18)
N1A—C1A—C2A	121.03 (19)	C3B—C4B—C6B	120.72 (19)
C1A—C2A—C3A	121.3 (2)	C5B—C4B—C6B	120.77 (19)
C2A—C3A—C4A	117.04 (19)	N1B—C5B—C4B	123.12 (19)
N3A—C3A—C2A	121.5 (2)	N1B—C7B—N2B	113.19 (19)
N3A—C3A—C4A	121.5 (2)	O1B—C8B—C9B	121.33 (19)
C3A—C4A—C6A	121.1 (2)	N2B—C8B—C9B	116.39 (18)
C3A—C4A—C5A	118.23 (19)	O1B—C8B—N2B	122.1 (2)
C5A—C4A—C6A	120.7 (2)	C10B—C9B—C14B	119.4 (2)
N1A—C5A—C4A	123.08 (19)	C8B—C9B—C10B	119.14 (19)
N1A—C7A—N2A	113.36 (16)	C8B—C9B—C14B	121.05 (19)
O1A—C8A—N2A	121.8 (2)	C9B—C10B—C11B	120.6 (2)
N2A—C8A—C9A	116.71 (18)	C10B—C11B—C12B	120.1 (3)
O1A—C8A—C9A	121.54 (18)	C11B—C12B—C13B	119.8 (3)
C8A—C9A—C14A	118.39 (18)	C12B—C13B—C14B	121.3 (2)
C10A—C9A—C14A	119.6 (2)	C9B—C14B—C13B	118.78 (19)
C8A—C9A—C10A	121.9 (2)	C9B—C14B—C15B	121.88 (18)
C9A—C10A—C11A	120.5 (2)	C13B—C14B—C15B	119.32 (18)
C10A—C11A—C12A	119.9 (2)	O2B—C15B—C14B	117.17 (17)
C11A—C12A—C13A	120.4 (2)	O2B—C15B—O3B	125.5 (2)
C12A—C13A—C14A	120.6 (2)	O3B—C15B—C14B	117.32 (19)
C9A—C14A—C15A	119.71 (19)	C2B—C1B—H1BA	119.00
C13A—C14A—C15A	121.2 (2)	N1B—C1B—H1BA	119.00
C9A—C14A—C13A	119.0 (2)	C1B—C2B—H2BA	119.00
O3A—C15A—C14A	117.7 (2)	C3B—C2B—H2BA	119.00
O2A—C15A—O3A	126.7 (2)	C4B—C5B—H5BA	118.00
O2A—C15A—C14A	115.5 (2)	N1B—C5B—H5BA	118.00
C2A—C1A—H1AA	119.00	C4B—C6B—H6BA	109.00
N1A—C1A—H1AA	119.00	C4B—C6B—H6BC	110.00
C3A—C2A—H2AA	119.00	H6BA—C6B—H6BB	109.00
C1A—C2A—H2AA	119.00	C4B—C6B—H6BB	109.00
N1A—C5A—H5AA	118.00	H6BB—C6B—H6BC	110.00
C4A—C5A—H5AA	119.00	H6BA—C6B—H6BC	110.00
C4A—C6A—H6AC	109.00	N1B—C7B—H7BB	109.00
H6AA—C6A—H6AB	109.00	N2B—C7B—H7BA	109.00
C4A—C6A—H6AB	110.00	N1B—C7B—H7BA	109.00
C4A—C6A—H6AA	109.00	H7BA—C7B—H7BB	108.00
H6AA—C6A—H6AC	110.00	N2B—C7B—H7BB	109.00
H6AB—C6A—H6AC	109.00	C9B—C10B—H10B	120.00
H7AA—C7A—H7AB	108.00	C11B—C10B—H10B	120.00
N2A—C7A—H7AB	109.00	C12B—C11B—H11B	120.00
N2A—C7A—H7AA	109.00	C10B—C11B—H11B	120.00
N1A—C7A—H7AA	109.00	C11B—C12B—H12B	120.00
N1A—C7A—H7AB	109.00	C13B—C12B—H12B	120.00
C11A—C10A—H10A	120.00	C12B—C13B—H13B	119.00
C9A—C10A—H10A	120.00	C14B—C13B—H13B	119.00
C10A—C11A—H11A	120.00		
			
C5A—N1A—C1A—C2A	0.8 (3)	C9A—C10A—C11A—C12A	0.0 (4)
C7A—N1A—C1A—C2A	178.52 (18)	C10A—C11A—C12A—C13A	1.0 (4)
C1A—N1A—C5A—C4A	−1.7 (3)	C11A—C12A—C13A—C14A	−1.6 (4)
C7A—N1A—C5A—C4A	−179.45 (18)	C12A—C13A—C14A—C15A	−175.7 (2)
C1A—N1A—C7A—N2A	112.8 (2)	C12A—C13A—C14A—C9A	1.0 (3)
C5A—N1A—C7A—N2A	−69.5 (2)	C9A—C14A—C15A—O2A	−51.8 (3)
C8A—N2A—C7A—N1A	−88.4 (2)	C13A—C14A—C15A—O3A	−52.1 (3)
C7A—N2A—C8A—O1A	15.1 (3)	C9A—C14A—C15A—O3A	131.2 (2)
C7A—N2A—C8A—C9A	−165.31 (18)	C13A—C14A—C15A—O2A	125.0 (2)
C7B—N1B—C5B—C4B	−178.6 (2)	N1B—C1B—C2B—C3B	0.1 (3)
C1B—N1B—C7B—N2B	97.4 (2)	C1B—C2B—C3B—N3B	179.6 (2)
C5B—N1B—C7B—N2B	−85.4 (2)	C1B—C2B—C3B—C4B	−1.0 (3)
C5B—N1B—C1B—C2B	1.1 (3)	N3B—C3B—C4B—C5B	−179.8 (2)
C7B—N1B—C1B—C2B	178.3 (2)	N3B—C3B—C4B—C6B	2.6 (3)
C1B—N1B—C5B—C4B	−1.4 (3)	C2B—C3B—C4B—C5B	0.8 (3)
C8B—N2B—C7B—N1B	−83.4 (2)	C2B—C3B—C4B—C6B	−176.8 (2)
C7B—N2B—C8B—O1B	4.4 (3)	C3B—C4B—C5B—N1B	0.4 (3)
C7B—N2B—C8B—C9B	−179.84 (19)	C6B—C4B—C5B—N1B	178.0 (2)
N1A—C1A—C2A—C3A	1.5 (3)	O1B—C8B—C9B—C10B	115.5 (3)
C1A—C2A—C3A—N3A	177.5 (2)	O1B—C8B—C9B—C14B	−57.2 (3)
C1A—C2A—C3A—C4A	−2.8 (3)	N2B—C8B—C9B—C10B	−60.3 (3)
N3A—C3A—C4A—C5A	−178.40 (19)	N2B—C8B—C9B—C14B	127.0 (2)
C2A—C3A—C4A—C6A	−178.4 (2)	C8B—C9B—C10B—C11B	−171.8 (2)
C2A—C3A—C4A—C5A	2.0 (3)	C14B—C9B—C10B—C11B	1.1 (4)
N3A—C3A—C4A—C6A	1.3 (3)	C8B—C9B—C14B—C13B	171.6 (2)
C6A—C4A—C5A—N1A	−179.4 (2)	C8B—C9B—C14B—C15B	−10.1 (3)
C3A—C4A—C5A—N1A	0.3 (3)	C10B—C9B—C14B—C13B	−1.2 (3)
O1A—C8A—C9A—C10A	111.7 (2)	C10B—C9B—C14B—C15B	177.2 (2)
N2A—C8A—C9A—C14A	116.8 (2)	C9B—C10B—C11B—C12B	−0.4 (4)
O1A—C8A—C9A—C14A	−63.6 (3)	C10B—C11B—C12B—C13B	−0.4 (4)
N2A—C8A—C9A—C10A	−67.9 (3)	C11B—C12B—C13B—C14B	0.4 (4)
C14A—C9A—C10A—C11A	−0.5 (3)	C12B—C13B—C14B—C9B	0.4 (3)
C8A—C9A—C14A—C13A	175.42 (19)	C12B—C13B—C14B—C15B	−178.0 (2)
C8A—C9A—C14A—C15A	−7.8 (3)	C9B—C14B—C15B—O2B	−38.6 (3)
C10A—C9A—C14A—C13A	0.0 (3)	C9B—C14B—C15B—O3B	142.6 (2)
C10A—C9A—C14A—C15A	176.8 (2)	C13B—C14B—C15B—O2B	139.7 (2)
C8A—C9A—C10A—C11A	−175.8 (2)	C13B—C14B—C15B—O3B	−39.1 (3)

### Hydrogen-bond geometry (Å, º)

*Cg*1 and *Cg*2 are the centroids of the C9B–C14B and N1B/C1B–C5B rings, respectively.

**Table:**

*D*—H···*A*	*D*—H	H···*A*	*D*···*A*	*D*—H···*A*
N2*A*—H2*N*2···O2*B*	0.88 (2)	2.03 (2)	2.897 (2)	173 (2)
N3*A*—H2*N*3···O3*B*^i^	0.93 (3)	1.95 (3)	2.864 (3)	168 (2)
N3*A*—H1*N*3···O3*A*^ii^	0.90 (2)	2.01 (3)	2.864 (3)	157 (2)
N2*B*—H1*N*2···O2*A*	0.90 (3)	2.12 (3)	3.006 (3)	171 (3)
N3*B*—H4*N*3···O3*B*^iii^	0.89 (2)	2.01 (3)	2.887 (3)	167 (2)
N3*B*—H3*N*3···O3*A*^iv^	0.95 (3)	2.03 (3)	2.935 (3)	161 (3)
O1*W*—H1*W*1···O2*B*	0.87	1.87	2.681 (3)	155
O1*W*—H2*W*1···O2*A*^v^	0.86	1.81	2.581 (3)	147
C1*A*—H1*AA*···O1*B*^vi^	0.93	2.53	3.355 (3)	147
C5*A*—H5*AA*···O1*W*	0.93	2.27	3.083 (3)	146
C7*A*—H7*AA*···O1*W*	0.97	2.47	3.139 (3)	126
C7*A*—H7*AB*···O1*B*^vi^	0.97	2.40	3.336 (3)	162
C1*B*—H1*BA*···O1*A*^v^	0.93	2.20	3.053 (3)	152
C2*A*—H2*AA*···*Cg*1^i^	0.93	2.95	3.831 (2)	158
C11*B*—H11*B*···*Cg*2^vii^	0.93	2.94	3.721 (3)	142

Symmetry codes: (i) *x*−1/2, *y*+1/2, *z*; (ii) *x*−1/2, *y*−1/2, *z*; (iii) *x*, −*y*, *z*−1/2; (iv) *x*, −*y*+1, *z*−1/2; (v) −*x*+1/2, *y*−1/2, −*z*+1/2; (vi) −*x*+1/2, *y*+1/2, −*z*+1/2; (vii) −*x*+1, *y*, −*z*+1/2.
